# A high-N00N output of harmonically driven cavity QED

**DOI:** 10.1038/s41598-019-49465-7

**Published:** 2019-11-14

**Authors:** Yusef Maleki, Aleksei M. Zheltikov

**Affiliations:** 1 Department of Physics and Astronomy, Texas A&M University, College Station, Texas 77843-4242 USA; 2 Physics Department, International Laser Center, M.V. Lomonosov Moscow State University, Moscow, 119992 Russia; 3 Russian Quantum Center, ul. Novaya 100, Skolkovo, Moscow Region, 143025 Russia; 4 National University of Science and Technology “MISiS”, Leninskii pr. 4, Moscow, 119049 Russia

**Keywords:** Quantum optics, Quantum information, Quantum mechanics, Quantum metrology

## Abstract

A harmonically driven cavity QED system consisting of two cavities and a two-level qubit is shown to enable the generation of a vast class of maximally entangled states suitable for measurements with a Heisenberg-limit precision. As one of its modalities, this system can serve as a quantum beam splitter, converting an |*N*〉 ⊗ |0〉 input into a maximally entangled N00N state (|*N*〉 ⊗ |0〉  +  |0〉 ⊗ |*N*〉)/$\sqrt{{\bf{2}}}$ at its output. A network of such quantum beam splitters is shown to provide a source of multimode N00N-type entanglement.

## Introduction

Quantum physics leads us to rethink and redefine the limits of precision in a physical measurement. When applied to an estimation of a parameter $\phi$ by sampling a system *N* times, suitably tailored quantum states can overcome the classical shot-noise limit (SNL) of parameter-estimation error, $\Delta \phi \propto 1/\sqrt{N}$, taking it to a new level of precision, limited only by the Heisenberg limit, $\Delta \phi \propto 1/N$^1,2^.

Maximally entangled quantum states $(|N\rangle \otimes |0\rangle +|0\rangle \otimes |N\rangle )/\sqrt{2}$, referred to as N00N states^3–5^, stand out as a conceptually and methodologically important example of quantum entanglement that enables measurements with a Heisenberg-limit (HL) precision^1,5^. Generation of such states in a realistic experimental setting is, however, anything but trivial. As a result, the values of *N* attainable with the existing experimental methods of N00N-state generation^6^ are rather low compared to the *N* values that have been achieved for other prominent classes of quantum states broadly used in photonic quantum technologies. Indeed, while coherent states with *N* $\simeq$ 100, *N* = 15 Fock states, and *N* = 10 Greenberger–Horne–Zeilinger (GHZ) states are all experimentally attainable^7–9^, N00N states with *N* > 5 photons are still beyond the capabilities of currently existing technologies.

As a promising direction toward practical sources of N00N-type states for HL measurements, a quantum interferometer with a nonlinear phase shift in one of its arms has been shown^10,11^ to provide a quantum beam-splitter-type scheme whereby an $|N\rangle \otimes |0\rangle$ state at the input can be converted into a N00N-state output. Implementation of this elegant concept in an all-optical format calls for systems with enhanced optical nonlinearities. Recent experiments suggest that a breakthrough to this much-needed nonlinearity enhancement may be achieved in systems with electromagnetically induced transparency (EIT)^12–14^ or resonance-enhanced Kerr effect^15,16^ inside an atom trap^14^, an optical cavity^17^, or a hollow waveguide^13,15,16^.

Here, in search for the ways to enhance the performance of a quantum beam splitter as source of quantum entanglement, we examine a generic harmonically driven cavity QED system consisting of two cavities and a two-level qubit. We show that such a system offers an interesting extension of the quantum beam-splitter concept that does not require an all-optical nonlinearity for multipartite entanglement generation. Instead, it is a periodic modulation of the parameters of the two-cavity–qubit system, that enables a control over the phase of quantum interference, needed to steer its output toward a N00N-type state. Effective-Hamiltonian analysis of such a system reveals physically insightful parallels with the Hamiltonians of both an ordinary beam splitter and a Kerr-nonlinearity-enabled quantum beam splitter. A network of such harmonically driven two-cavity–qubit quantum beam splitters is shown to provide a source of multimode N00N-type entanglement.

## The Magic of a Quantum Beam Splitter

We start by briefly examining a generic ordinary beam splitter as an important point of reference. When fed with a single-photon state $|1\rangle$ and a vacuum state $|0\rangle$ through its two input ports, a standard beam splitter will deliver $(|1\rangle \otimes |0\rangle +|0\rangle \otimes |1\rangle )/\sqrt{2}$, that is, a N00N state with *N* = 1, at its output. However, when a single-photon state $|1\rangle$ at the input of a beam splitter is replaced with a larger *N*-photon state $|N\rangle$, the state produced as an output of the beam splitter cannot be reduced to a N00N state.

With an ordinary beam splitter being unable to deliver a pure N00N-state output, we come to realize that it takes a kind of a “magic” beam splitter^18^ to generate N00N states with *N* > 1, from the input $|N\rangle \otimes |0\rangle$ state. The Kerr-type optical nonlinearity has been shown^10,11^ to lend such a magic touch, providing a physical mechanism whereby a suitable nonlinear phase shift can be induced in one of the arms of a quantum interferometer, referred to as a quantum beam splitter (QBS), enabling N00N-state generation at its output.

In this work, in search for the ways to enhance the field–matter coupling – the key factor in control of the QBS performance – beyond the limitations of the purely optical Kerr effect, we explore the cavity-QED extension of the QBS concept. This approach, as will be shown below in this paper, opens the routes toward enhanced QBS performance due to a much stronger field–matter coupling attainable in the cavity QED setting. As a specific example of a cavity QED system, we consider a two-level qubit coupled to two cavities. The effective Hamiltonian of such a system, as we show in this work, can be written as

1 $${H}_{{\rm{eff}}}=i\kappa ({a}_{1}{a}_{2}^{\dagger }-{a}_{2}{a}_{1}^{\dagger }){\sigma }_{z},$$

where $\kappa$ is the cavity–qubit coupling coefficient, ${a}_{i}^{\dagger }$ and *a*_*i*_ are the photon creation and annihilation operators for the *i*th cavity field, and ${\sigma }_{z}=|e\rangle \langle e|-|g\rangle \langle g|$, $|e\rangle$ and $|g\rangle$ being the two states of the two-level qubit.

The Hamiltonian of Eq. (1) is in many ways instructive as it bears a pleasing resemblance of both – the Hamiltonian of an ordinary beam splitter and the Hamiltonian of a quantum beam splitter based on the Kerr effect. We articulate that the scope of Eq. (1) goes way beyond a specific cavity-QED QBS implementation, as this equation, in fact, sets up the effective Hamiltonian for a generic QBS source of N00N states, converting an $|N\rangle |0\rangle$ input into a N00N-state output. We therefore intend to focus first on the properties of the Hamiltonian (1) as a generic source of N00N states, providing its consistent derivation later, where Eq. (1) will be shown to define the effective Hamitonian for a specific, two-cavity–qubit cavity-QED system.

When used jointly with the Heisenberg evolution equation, the Hamiltonian (1) leads to the following evolution equations for the fields:

2 $$\begin{array}{rcl}{\dot{a}}_{1}(t) & = & -\kappa {a}_{2}(t){\sigma }_{z},\\ {\dot{a}}_{2}(t) & = & \kappa {a}_{1}(t){\sigma }_{z}.\end{array}$$

The solution to Eq. (2) is

3 $$\begin{array}{rcl}{a}_{1} & = & {a}_{1}(0)\,\cos (\kappa t)-{a}_{2}(0)\,\sin (\kappa t){\sigma }_{z},\\ {a}_{2} & = & {a}_{2}(0)\,\cos (\kappa t)+{a}_{1}(0)\,\sin (\kappa t){\sigma }_{z}.\end{array}$$

With $\kappa t=\pi /4$, Eq. (3) give

$$\begin{array}{rcl}{a}_{1} & = & \frac{1}{\sqrt{2}}({a}_{1}(0)-{a}_{2}(0){\sigma }_{z}),\\ {a}_{2} & = & \frac{1}{\sqrt{2}}({a}_{2}(0)+{a}_{1}(0){\sigma }_{z}).\end{array}$$

**Figure 1:**
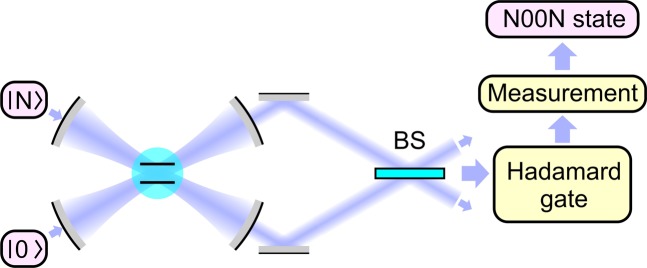
Generation of N00N states in a cavity QED system consisting of a qubit and two cavities: M, mirrors; BS, 50:50 beam splitter. Quantum states $|0\rangle$ and $|N\rangle$ enter the system through the two input ports. The output, produced at $\kappa t=\pi /4$, is transmitted through a 50:50 beam splitter and an Hadamard gate and is detected with a measuring device to yield a N00N state.

These fields are then transmitted through a 50:50 beam splitter (see Fig. 1), providing a two-mode unitary transformation $U=\exp [\,-\,\pi /4({a}_{1}^{\dagger }{a}_{2}-{a}_{2}^{\dagger }{a}_{1})]$ and yielding

$$\begin{array}{rcl}{a}_{1} & \to  & \frac{1}{\sqrt{2}}({a}_{1}+{a}_{2}),\\ {a}_{2} & \to  & \frac{1}{\sqrt{2}}({a}_{2}-{a}_{1}).\end{array}$$

An overall transformation of the input fields provided by the two-cavity–qubit system and the 50:50 beam splitter is

4 $${a}_{1}\to {a}_{1}(0)|e\rangle \langle e|+{a}_{2}(0)|g\rangle \langle g|,$$

5 $${a}_{2}\to {a}_{2}(0)|e\rangle \langle e|-{a}_{1}(0)|g\rangle \langle g|$$

These transformations are central to understanding the entanglement-generation capability of our qubit–two-cavity system. Equations (4) and (5) dictate the following transformation of a $|\psi \rangle =|N\rangle |0\rangle (|g\rangle +|e\rangle )/\sqrt{2}$ input: $|\psi \rangle \to \frac{1}{\sqrt{2}}(|N\rangle |0\rangle |e\rangle +|0\rangle |N\rangle |g\rangle )/\sqrt{2}$.

With an Hadamard gate applied to the qubit (Fig. 1), e.g., by an external *π*/2 pulse, the output state becomes $[(|N\rangle |0\rangle +|0\rangle |N\rangle )|e\rangle +(|N\rangle |0\rangle -|0\rangle |N\rangle )|g\rangle ]/2$. A measurement of the qubit state will then yield a N00N state. Specifically, the detection of the qubit in its ground and excited states $|g\rangle$ and $|e\rangle$ gives the N00N states $(|N\rangle |0\rangle -|0\rangle |N\rangle )/\sqrt{2}$ and $(|N\rangle |0\rangle +|0\rangle |N\rangle )/\sqrt{2}$, respectively. Either of these output N00N states will enable parameter estimation with the HL precision.

In an earlier study, Yang *et al*.^19^ have proposed an elegant circuit QED source of enhanced quantum entanglement based on a four-level qubit coupled to two cavities. The cavity QED scheme considered in this work, on the other hand, employs a two-level qubit, which helps avoid difficulties related to the second-order detuning term in the effective Hamiltonian, which tends to weaken qubit–cavity coupling, making the entangled-state output prone to decoherence.

## Enhanced Entanglement Beyond N00N States

In this section, we will demonstrate that the ability of the considered two-cavity–qubit system to generate enhanced quantum entanglement is not limited to a N00N-state output, but extends to a much broader class of quantum states. To this end, we take the input of the first cavity in the form of a coherent state^20^, $|\alpha \rangle ={e}^{-\frac{|\alpha {|}^{2}}{2}}\,{\sum }_{n=0}^{\infty }\,\frac{{\alpha }^{n}}{\sqrt{n!}}|n\rangle$, *α* being an arbitrary complex number. Then, with $|0\rangle$ at the second input port, the initial state of the system is $|\psi \rangle =|\alpha \rangle |0\rangle \,(|g\rangle +|e\rangle )/\sqrt{2}$. Quantum evolution of this state dictated by Eqs (4) and (5) yields $(|\alpha \rangle |0\rangle |e\rangle +|0\rangle |\alpha \rangle |g\rangle )/\sqrt{2}$. Applying an Hadamard gate to this state and performing a measurement on the qubit, we arrive at

6 $$|{\phi }^{\pm }\rangle =\frac{1}{\sqrt{2\pm 2{e}^{-|\alpha {|}^{2}}}}(|\alpha \rangle |0\rangle \pm |0\rangle |\alpha \rangle ),$$

where, the minus or plus sign is taken depending on whether the qubit is detected in its ground or excited state. The state $|{\phi }^{+}\rangle$ is known to have important applications in quantum metrology^21^. With a squeezed state at its input, our system can yield a squeezed-state–vacuum entanglement.

For a general-form initial state $|\psi \rangle =|{\psi }_{1}\rangle |{\psi }_{2}\rangle \,(|g\rangle +|e\rangle )/\sqrt{2}$, with $|{\psi }_{1}\rangle ={\sum }_{n=0}^{{d}_{1}}\,{b}_{n}|n\rangle$ and $|{\psi }_{2}\rangle ={\sum }_{n=0}^{{d}_{2}}\,{c}_{n}|n\rangle$, our two-cavity–qubit system will create an antisymmetric or symmetric entangled-state output,

7 $$|{\psi }^{-}\rangle =\frac{1}{\sqrt{2-2|\langle {\psi }_{1}|{\psi }_{2}\rangle {|}^{2}}}(|{\psi }_{1}\rangle |{\psi }_{2}\rangle -|{\psi }_{2}\rangle |{\psi }_{1}\rangle ).$$

8 $$|{\psi }^{+}\rangle =\frac{1}{\sqrt{2+2|\langle {\psi }_{1}|{\psi }_{2}\rangle {|}^{2}}}(|{\psi }_{1}\rangle |{\psi }_{2}\rangle +|{\psi }_{2}\rangle |{\psi }_{1}\rangle ),$$

depending on whether the atom is detected in its ground or excited state.

With a properly tailored input, our cavity-QED QBS can also help generate superpositions composed of two coherent states ($$|\alpha \rangle $$ and $$|\beta \rangle $$), $$(|\alpha \rangle |\beta \rangle \pm |\beta \rangle |\alpha \rangle )/\sqrt{2\pm 2|\langle \alpha |\beta \rangle {|}^{2}}$$, two squeezed states ($$|{\xi }_{1}\rangle $$ and $$|{\xi }_{2}\rangle $$), $$(|{\xi }_{1}\rangle |{\xi }_{2}\rangle \pm $$$$|{\xi }_{2}\rangle |{\xi }_{1}\rangle )/\sqrt{2\pm 2|\langle {\xi }_{1}|{\xi }_{2}\rangle {|}^{2}}$$, or hybrid-entanglement combinations of Fock, coherent, and squeezed states, $$(|\alpha \rangle |{\xi }_{1}\rangle \pm $$$$|{\xi }_{1}\rangle |\alpha \rangle )/\sqrt{2\pm 2|\langle {\xi }_{1}|\alpha \rangle {|}^{2}}$$, $$(|\alpha \rangle |N\rangle \pm |N\rangle |\alpha \rangle )/\sqrt{2\pm 2|\langle N|\alpha \rangle {|}^{2}}$$, or $$(|{\xi }_{1}\rangle |N\rangle \pm |N\rangle |{\xi }_{1}\rangle )/$$$$\sqrt{2\pm 2|\langle N|{\xi }_{1}\rangle {|}^{2}}$$. Such states can help overcome the SNL in quantum metrology and promise interesting options for quantum information processing.

The entanglement of $|{\psi }^{\pm }\rangle$ can be quantified in terms of the concurrence^22^, defined as

9 $$C=|\langle \psi |{\sigma }_{y}\otimes {\sigma }_{y}|{\psi }^{\ast }\rangle |,$$

where *σ*_*y*_ is the spin flip operator and $|{\psi }^{\ast }\rangle$ is the complex conjugate of $|\psi \rangle$. When applied to $|{\psi }^{\pm }\rangle$, Eq. (9) gives

10 $$C=\frac{1-|\langle {\psi }_{1}|{\psi }_{2}\rangle {|}^{2}}{1\pm |\langle {\psi }_{1}|{\psi }_{2}\rangle {|}^{2}},$$

with the plus and minus signs taken for $|{\psi }^{+}\rangle$ and $|{\psi }^{-}\rangle$, respectively. Notably, while the maximum entanglement of $|{\psi }^{-}\rangle$ is achieved with any input state, $|{\psi }^{+}\rangle$ is maximally entangled if and only if the input states are orthogonal states, with $\langle {\psi }_{1}|{\psi }_{2}\rangle =0$. The maximum entanglement of the $|{\psi }^{-}\rangle$ output makes our two-cavity–qubit QBS a powerful resource not only for quantum sensing and parameter estimation, but also for quantum computing and information processing.

As an important example, with an $|N\rangle |M\rangle$ state at its input, our QBS will produce a maximally entangled state $(|N\rangle |M\rangle \pm |M\rangle |N\rangle )/\sqrt{2}$, as its output. With $M=0$, this output reduces to a N00N state.

## The Effective Hamiltonian

In this section, we will consider specific examples of cavity-QED systems that can be described in terms of the effective Hamiltonian (1). Our analysis will be focused on a system consisting of two cavities with frequencies ${\omega }_{1}$ and ${\omega }_{2}$ and a two-level qubit whose levels are separated by an energy $\hslash {\omega }_{0}$. The qubit is coupled to the first and second cavities with coupling strengths *g*_1_ and *g*_2_. We will show below in this section that, when the cavity frequencies or the qubit–cavity coupling strengths in such a system are modulated periodically in time^23,24^, the quantum evolution of the system can be described in terms of the effective Hamiltonian (1), enabling the generation of a vast class of maximally entangled states suitable for measurements with an HL precision.

### Two-cavity–qubit system with a modulated coupling strength

We first consider a system of two cavities and a two-level qubit in which the coupling strengths *g*_1_ and *g*_2_ are harmonic functions of time, ${g}_{j}={g}_{0}\,\cos ({\upsilon }_{d}t+{\phi }_{j})$, $j=1,2$. Such harmonically modulated coupling terms are found in Hamiltonians describing superconducting-circuit magnetic field synthesizers^23,25,26^ and photonic resonator lattices exhibiting effective magnetic fields for photons^23,25,26^. We represent the full Hamiltonian of such a system as a sum $H={H}_{0}+{H}_{I}$ with

11 $${H}_{0}=\hslash \frac{{\omega }_{0}}{2}{\sigma }_{z}+\hslash {\omega }_{1}{a}_{1}^{\dagger }{a}_{1}+\hslash {\omega }_{2}{a}_{2}^{\dagger }{a}_{2}$$

and

12 $${H}_{I}=2\hslash {g}_{0}\,\mathop{\sum }\limits_{j=0}^{2}\,\cos ({\upsilon }_{d}t+{\phi }_{j})\,({\sigma }^{+}{a}_{j}+{a}_{j}^{\dagger }{\sigma }^{-}).$$

We set, with an appropriate choice of the system of units, $\hslash =1$ and assume the free Hamiltonian defined as ${V}_{0}=\frac{\omega }{2}{\sigma }_{z}+\omega {a}_{1}^{\dagger }{a}_{1}+\omega {a}_{2}^{\dagger }{a}_{2}$. In the frame rotating with *V*_0_, the Hamiltoninan can be given as

13 $${H}_{I}={U}_{0}(t)H{U}_{0}^{-1}(t)-{V}_{0},$$

where ${U}_{0}(t)=\exp [\,-\,i{V}_{0}t]$ is the unitary evolution operator. Noting that $[{H}_{0},{V}_{0}]=0$, the resultant Hamiltonian reduces to

14 $${H}_{I}={\delta }_{0}{\sigma }_{z}/2+{\delta }_{1}{a}_{1}^{\dagger }{a}_{1}+{\delta }_{2}{a}_{2}^{\dagger }{a}_{2}+2{g}_{0}\,\mathop{\sum }\limits_{j=1}^{2}\,\cos ({\upsilon }_{d}t+{\phi }_{j})\,({\sigma }^{+}{a}_{j}+{a}_{j}^{\dagger }{\sigma }^{-}),$$

where $${\delta }_{0}={\omega }_{0}-\omega $$, $${\delta }_{1}={\omega }_{1}-\omega $$ and $${\delta }_{2}={\omega }_{2}-\omega $$.

Equation (14) can be rewritten as

$$\begin{array}{rcl}{H}_{I} & = & {\delta }_{0}{\sigma }_{z}/2+{\delta }_{1}{a}_{1}^{\dagger }{a}_{1}+{\delta }_{2}{a}_{2}^{\dagger }{a}_{2}++{g}_{0}\,\mathop{\sum }\limits_{j=1}^{2}\,[({\sigma }^{+}{a}_{j}+{a}_{j}^{\dagger }{\sigma }^{-}){e}^{i{\phi }_{j}}]{e}^{i{\upsilon }_{d}t}\\  &  & +\,[({\sigma }^{+}{a}_{j}+{a}_{j}^{\dagger }{\sigma }^{-}){e}^{-i{\phi }_{j}}]{e}^{-i{\upsilon }_{d}t}.\end{array}$$

Defining an operator ${h}_{j}={g}_{0}({\sigma }^{+}{a}_{j}+{a}_{j}^{\dagger }{\sigma }^{-}){e}^{i{\phi }_{j}}$, we reduce *H*_*I*_ to a Floquet Hamiltonian^24,27–29^, ${H}_{I}={\delta }_{0}{\sigma }_{z}/2+{\delta }_{1}{a}_{1}^{\dagger }{a}_{1}+{\delta }_{2}{a}_{2}^{\dagger }{a}_{2}++\,{\sum }_{j=1}^{2}\,{h}_{j}^{\dagger }{e}^{i{\upsilon }_{d}t}+{h}_{j}{e}^{-i{\upsilon }_{d}t}$. Considering the condition ${\upsilon }_{d}\gg \sqrt{N}{g}_{0}$ with *N* being the number of the input photons, we can find the effective Floquet Hamiltonian by adiabatically eliminating the fast oscillating terms^24^. Thus, using the commutation relations $[{h}_{j},{h}_{j}^{\dagger }]=0$ we find for the effective Hamiltonian^24,27–29^

15 $${H}_{{\rm{eff}}}={\delta }_{0}{\sigma }_{z}/2+{\delta }_{1}{a}_{1}^{\dagger }{a}_{1}+{\delta }_{2}{a}_{2}^{\dagger }{a}_{2}+i\frac{2{g}_{0}^{2}}{{\upsilon }_{d}}\,\sin ({\phi }_{1}-{\phi }_{2})\,({a}_{1}{a}_{2}^{\dagger }-{a}_{2}{a}_{1}^{\dagger }){\sigma }_{z}.$$

As can be seen from Eq. (15), the coupling term of the effective Hamiltonian is controlled by the phase difference ${\phi }_{1}-{\phi }_{2}$. To maximize this term, we choose ${\phi }_{1}-{\phi }_{2}=\pi /2$. Equation (15) then becomes

16 $${H}_{{\rm{eff}}}={\delta }_{0}{\sigma }_{z}/2+{\delta }_{1}{a}_{1}^{\dagger }{a}_{1}+{\delta }_{2}{a}_{2}^{\dagger }{a}_{2}+i\kappa {\sigma }_{z}({a}_{1}{a}_{2}^{\dagger }-{a}_{2}{a}_{1}^{\dagger }),$$

where $$\kappa =\frac{2{g}_{0}^{2}}{{\upsilon }_{d}}$$.

Note that *σ*_*z*_ commutes with the rest of the Hamiltonian, thus, on a resonance condition on the cavities, Eq. (16) reduces to the sought-for effective Hamiltonian of Eq. (1).

### Two-cavity–qubit system with modulated cavity frequencies

We now assume that the coupling strength in our two-cavity–qubit QBS is constant, but the cavity frequencies are harmonic functions of time, ${\omega }_{j}(t)=\nu +\Delta \,\sin ({\nu }_{d}t-{\phi }_{j})$, *j* = 1, 2. The Hamiltonian of the system is then written as

17 $$\begin{array}{rcl}H & = & \hslash \frac{{\omega }_{0}}{2}{\sigma }_{z}+\hslash {\omega }_{1}(t){a}_{1}^{\dagger }{a}_{1}+\hslash {\omega }_{2}(t){a}_{2}^{\dagger }{a}_{2}\\  &  & +\,\hslash g({\sigma }^{+}{a}_{1}+{a}_{1}^{\dagger }{\sigma }^{-}+{\sigma }^{+}{a}_{2}+{a}_{2}^{\dagger }{\sigma }^{-}).\end{array}$$

On a resonance, ${\omega }_{0}=\nu$, we find for *H*_*I*_ in the rotating-wave approximation

$${H}_{I}=\hslash g{\sigma }^{+}({\hat{a}}_{1}{e}^{i\zeta \cos ({\nu }_{d}t-{\phi }_{1})}+{\hat{a}}_{2}{e}^{i\zeta \cos ({\nu }_{d}t-{\phi }_{2})})+h.c.,$$

where $$\zeta =\Delta /{\nu }_{d}$$.

We can now use the identity ${e}^{i\zeta \cos ({\nu }_{d}t+{\phi }_{j})}=\mathop{\sum }\limits_{n=-\infty }^{\infty }\,{J}_{n}(\zeta ){e}^{in({\nu }_{d}t+{\phi }_{j})}$, ${J}_{n}(\zeta )$ being the *n*th-order Bessel function of the first kind, to reduce *H*_*I*_ to a Floquet Hamiltonian, ${H}_{I}={H}_{0}+{\sum }_{n=1}^{\infty }\,{H}_{n}{e}^{in{\nu }_{d}t}$, with

18 $${H}_{0}=\hslash g{J}_{0}(\zeta )\,({\sigma }^{+}({\hat{a}}_{1}+{\hat{a}}_{2})+({\hat{a}}_{1}^{\dagger }+{\hat{a}}_{2}^{\dagger }){\sigma }^{-})$$

and

19 $${H}_{n}=\hslash g{i}^{n}{J}_{n}(\zeta )[({\sigma }^{+}{\hat{a}}_{1}+{(-1)}^{n}{\hat{a}}_{1}^{\dagger }{\sigma }^{-}){e}^{in{\phi }_{1}}+({\sigma }^{+}{\hat{a}}_{2}+{(-1)}^{n}{\hat{a}}_{2}^{\dagger }{\sigma }^{-}){e}^{in{\phi }_{2}}].$$

The interaction Hamiltonian of this form leads to an effective Hamiltonian^28,29^

20 $${H}_{{\rm{eff}}}={H}_{0}+\mathop{\sum }\limits_{n=1}^{\infty }\,[{H}_{n},{H}_{-n}]/(n\hslash {\nu }_{d}).$$

Combining Eqs (18)–(20), we derive^24,27–29^

21 $${H}_{{\rm{eff}}}=\hslash g{J}_{0}(\zeta )\,({\sigma }^{+}({\hat{a}}_{1}+{\hat{a}}_{2})+h.c.)+i\hslash \Omega ({\hat{a}}_{1}^{\dagger }{\hat{a}}_{2}-{\hat{a}}_{1}{\hat{a}}_{2}^{\dagger }){\sigma }_{z}.$$

where $$\Omega ={g}^{2}\chi /{\nu }_{d}$$ and $$\chi ={\sum }_{n=1}^{\infty }\,2{J}_{n}{(\zeta )}^{2}\,\sin (n({\phi }_{1}-{\phi }_{2}))/n$$.

Choosing $\zeta$ such that ${J}_{0}(\zeta )\approx 0$ ($\zeta \approx 2.405$), we find

22 $${H}_{{\rm{eff}}}=i\hslash \Omega ({\hat{a}}_{1}^{\dagger }{\hat{a}}_{2}-{\hat{a}}_{1}{\hat{a}}_{2}^{\dagger }){\sigma }_{z}$$

The effective coupling coefficient $\Omega$ can be maximized with an appropriate choice of ${\phi }_{1}-{\phi }_{2}$. Specifically, ${\phi }_{1}-{\phi }_{2}\approx \pi /3$ leads to $\chi \approx 0.628$. Qubit–resonator coupling as strong as $\simeq$100 MHz can be achieved for a flux qubit coupled to a pair of superconductor cavities^30,31^, providing $\kappa \simeq \Omega \simeq 10-100\,{\rm{MHz}}$. Specifically, with ${\upsilon }_{d}=8{g}_{0}$ and ${g}_{0}\simeq 50\,{\rm{MHz}}$ in Eq. (16), we arrive at $\kappa \simeq 12.5\,{\rm{MHz}}$, which is well above the decoherence rates of the cavities and the qubit, typically estimated as $\simeq$1 MHz^32^.

## Multimode Entanglement

**Figure 2:**
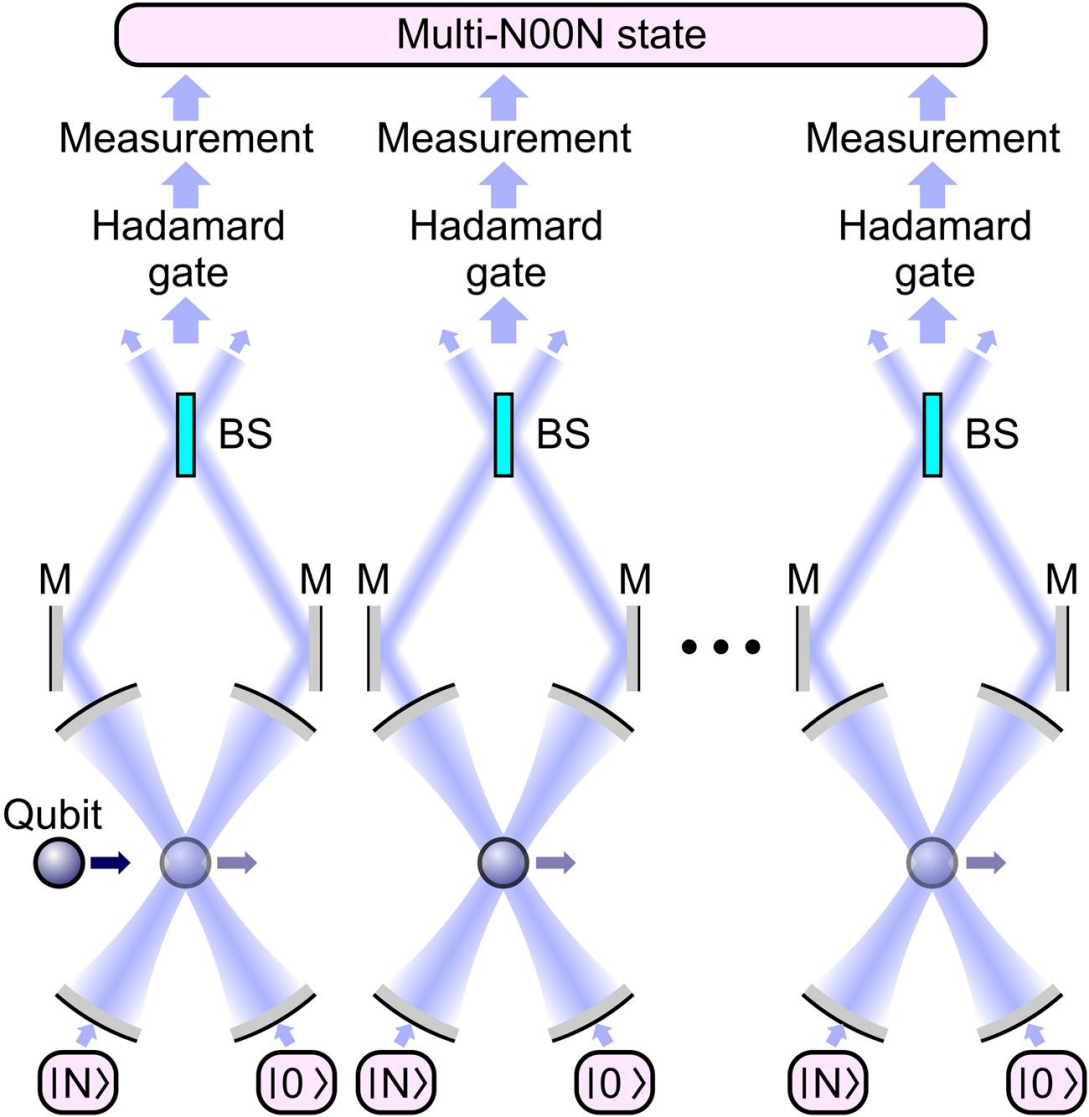
Generation of multi-N00N states in a network of quantum beam splitters: M, mirrors; BS, 50:50 beam splitters. A two-level qubit consecutively interacts with a series of cavity pairs. Photons created as a result of these interactions are sent to 50:50 beam splitters to yield a multi-N00N state output.

We are going to show now that, when connected into a network, cavity-QED QBS systems examined in the previous sections can help confront a challenge of multimode N00N-type entanglement generation. To this end, we consider an array of two-cavity units (Fig. 2) with a qubit set to consecutively interact with each pair of cavities within a time interval $t=\pi /(4{\kappa }_{0})$, where ${\kappa }_{0}=\kappa$ for a two-cavity–qubit system with a modulated coupling strength and ${\kappa }_{0}=\Omega$ for a two-cavity–qubit system with modulated cavity frequencies. Output cavity photons are sent to 50:50 beam splitters (Fig. 2).

When applied to an initial state $|\psi \rangle =|N,0\rangle \otimes |N,0\rangle \otimes \ldots \otimes |N,0\rangle \,(|g\rangle +|e\rangle )/\sqrt{2}$, this cavity QED array will yield $(|N,0\rangle \mathrm{...}|N,0\rangle |e\rangle +|0,N\rangle \ldots |0,N\rangle |g\rangle )/\sqrt{2}$ as its output. To illustrate, let us consider the input state with two cavity pairs as $|\psi \rangle =|N,0\rangle \otimes |N,0\rangle \otimes (|g\rangle +|e\rangle )/\sqrt{2}$. First, the qubit interacts with the first cavity pair resulting in $(|N,0\rangle |e\rangle +|0,N\rangle |g\rangle )/\sqrt{2}\otimes |N,0\rangle$. Then, the qubit interacts with the second cavity pair which prepares $(|N,0\rangle |N,0\rangle |e\rangle +|0,N\rangle |0,N\rangle |g\rangle )/\sqrt{2}$ as its output. With a *π*/2 pulse applied to the qubit and following a measurement, this output becomes a double-N00N state $(|N,0\rangle |N,0\rangle \pm |0,N\rangle |0,N\rangle )/\sqrt{2}$. Therefore, with the cavity QED array above, we can generate a multi-N00N state $(|N,0\rangle \ldots |N,0\rangle \pm |0,N\rangle \ldots |0,N\rangle )/\sqrt{2}$.

An array of *M* cavity pairs arranged in this scheme will then provide a phase resolution $\Delta \phi \propto 1/(MN)$. Achieving this level of Δ$\phi$ with ordinary N00N states would have required *MN* photons in each of the entangled modes. For large *M* and *N*, this requirement is difficult to fulfill in practice. To appreciate this, let us take $m=2$, for simplicity. In this case, the generated state reduces to the double N00N state $(|N,0\rangle |N,0\rangle \pm |0,N\rangle |0,N\rangle )/\sqrt{2}$ introduced in^33^. Applying the phase shift $\phi$ on the second and the fourth modes of the state, the phase shift of 2*N*$\phi$ is accumulated which results in $\Delta \phi \propto 1/(2N)$. This is equivalent to the phase estimation with a N00N state of the form $(|2N,0\rangle +|0,2N\rangle )/\sqrt{2}$^33^. Considering the fact that number states of more than *N* = 15 are feasible with the current technology^7,9^, preparing two pairs of *N* = 15 photons in cavity pairs can result in estimation with a phase resolution $\Delta \phi \propto 1/30$. Achieving such a big phase estimation number with an ordinary N00N state is far beyond the latest achievements with the current technology which is limited to a few photons. It is notable that our approach of double N00N state generation is different from the one introduced in^33^ in principle. In the setup proposed in^33^, *N*  +  2 operational steps is required for generating a double N00N state; however, in our system, we can produce a double N00N state in just a few operational steps, which is independent of the number of the photons, once the initial state is given. Furthermore, our structure is even capable of generating multi-N00N states that can facilitate quantum phase estimation techniques. That being said, we can generate a vast class of multi-mode entangled state by preparing each resonator in our specific state of interest. It is notable that in most of the recently introduced schemes, generating a N00N state requires a linear number of operations with *N*, which is usually greater than *N*^34,35^. The large operational steps required makes it challenging to generate such states experimentally, as decoherence is introduced to the system in each step of the operation. Thus, generation of multimode N00N state can be useful for overcoming this challenge.

## Conclusion

To summarize, we have shown that a harmonically driven cavity QED system consisting of two cavities and a two-level qubit enables the generation of a vast class of maximally entangled states suitable for measurements with an HL precision. As one of its modalities, this system can serve as a quantum beam splitter, converting an $|N\rangle \otimes |0\rangle$ input into a maximally entangled N00N state $(|N\rangle \otimes |0\rangle +|0\rangle \otimes |N\rangle )/\sqrt{2}$ at its output. A network of such quantum beam splitters is shown to provide a source of multimode N00N-type entanglement.
